# Disseminated Bartonellosis Involving the Kidney and Heart

**DOI:** 10.7759/cureus.113462

**Published:** 2026-07-27

**Authors:** Habib Behjatnia, Julio Alvarez-Cardona, Mario Madruga, Steve Carlan

**Affiliations:** 1 Internal Medicine, Orlando Regional Medical Center, Orlando, USA; 2 Infectious Diseases, Orlando Regional Medical Center, Orlando, USA; 3 Obstetrics, Orlando Regional Medical Center, Orlando, USA

**Keywords:** aortic valve vegetation, bartonella endocarditis, blood culture negative endocarditis, mitral valve vegetation, renal artery infarction

## Abstract

Blood culture-negative infective endocarditis poses considerable clinical and diagnostic challenges because of its atypical manifestations and the difficulty in identifying the responsible pathogens. The involvement of *Bartonella henselae,* a rare etiologic agent of blood culture-negative infective endocarditis, further complicates diagnosis and management. Dissemination of *Bartonella* across multiple organ systems is a recognized feature of the disease.

A 73-year-old female with a one-year history of prosthetic mitral valve replacement for mitral valve stenosis secondary to rheumatic fever presented with a one-year history of self-remitting fevers and worsening anemia. In the months preceding admission, she also experienced a cerebrovascular accident involving the left frontal lobe. She was known to care for feral cats, coming into close contact with them through feeding and combing their fur for fleas. CT (computed tomography) imaging on admission was concerning for a left renal infarction, prompting an embolic workup. The transesophageal echocardiogram was notable for irregular thickening of the bioprosthetic mitral valve and mobile, filamentous densities, suggestive of a vegetation. *Bartonella henselae* IgG titers were markedly elevated at 1:32,768, raising concerns for infective endocarditis secondary to *Bartonella henselae* with dissemination to the bone marrow and left kidney.

This report emphasizes the need for increased awareness, thorough diagnostic imaging, and thoughtful treatment approaches for a patient with widespread bartonellosis. Early identification of *Bartonella henselae* should be considered in blood culture-negative endocarditis cases, especially in patients with relevant epidemiological exposure.

## Introduction

*Bartonella henselae* is a fastidious, gram-negative bacterium best known for causing cat-scratch disease, bacillary angiomatosis, and a notable proportion of culture-negative endocarditis cases [1]. Because untreated *Bartonella* endocarditis carries a mortality rate of about 30%, early diagnosis and treatment are critical [2]. However, culture-negative endocarditis remains difficult to diagnose because of its indolent presentation and broad differential, which includes HACEK organisms, *Histoplasma capsulatum*, *Bartonella henselae*, and noninfectious conditions such as Libman-Sacks endocarditis [3]. Prior antibiotic use may further complicate diagnosis by causing pseudo-culture-negative endocarditis [4].

We report a case of *Bartonella henselae* culture-negative endocarditis with renal infarction, cerebrovascular accident, and bone marrow involvement, highlighting the organism’s insidious presentation and potential for delayed diagnosis.

To our knowledge, no prior English-language report describes concurrent *Bartonella* culture-negative endocarditis, renal infarction, cerebrovascular accident, and bone marrow involvement in a single patient.

## Case presentation

A 73-year-old woman presented with a one-year history of self-limited, low-grade cyclical fevers, typically worse in the evenings, that had intensified over the preceding three months.

Her history was notable for atrial fibrillation, patent foramen ovale, sick sinus syndrome, and rheumatic mitral stenosis. Five years before symptom onset, she underwent bioprosthetic mitral valve replacement, patent foramen ovale closure, and left atrial appendage ligation. A permanent pacemaker was implanted one year before symptom onset. She also had chronic normocytic anemia and polymyalgia rheumatica, treated with prednisone 1 mg daily. She frequently hunted in Georgia and consumed deer and wild boar. She also cared for two stray cats, combed them for fleas, and reported multiple scratches and bites. Her family and social history was otherwise negative.

Before admission, she underwent an evaluation for anemia, including a colonoscopy, which was unrevealing. In the preceding months, she had a cerebrovascular accident involving the left frontal lobe, adjacent insula, and left periventricular white matter, raising concern for an embolic source. Transthoracic echocardiography at that time showed no interatrial shunt or prosthetic valve abnormality, and she had no residual neurological deficits.

On admission, vital signs were stable. Laboratory testing showed severe normocytic anemia with a low reticulocyte percentage, inflammatory iron studies, hyponatremia, and mild renal dysfunction (Tables 1, 2).

**Table 1:** Complete blood cell assessment on admission * Abnormal result

Laboratory Tests	Units and Abbreviations	Normal Range	Patient Value
Hemoglobin*	Grams per deciliter (g/dL)	Women: 12.3–15.3	6.8
Hematocrit (%)*	%	Women: 36–46	20.2
White blood cell	Cells per microliter (cells/µL)	4,500–11,000	4,900
Platelet	Thousand per microliter (per µL)	100,000–400,000	148,000
Mean platelet volume	Femtoliter (fL)	7.0–13.0	9
Red blood cells	Million cells per microliter (cells/µL)	Women: 2–8	2.29
Mean corpuscular volume	Femtoliter (fL)	80–100	88.2
Mean corpuscular hemoglobin	Picograms per cell (pg)	27–33	29.7
Mean corpuscular hemoglobin concentration	Grams per deciliter (g/dL)	32–36	33.7
Red blood cell distribution width (%)	%	11–15	14.9
Iron/reticulocyte studies			
Hypochromia*	NA	NA	Moderate
Absolute reticulocyte count	%	0.5–2.5	1.8
Reticulocyte percentage	%	0.5–2.5	0.08
Reticulocyte RBC count*	Million cells per microliter (µL)	4.0–5.5	2.29

**Table 2:** Complete metabolic profile on admission * Abnormal result mg/dL = milligrams per deciliter; mEq/L = milliequivalents per liter; IU/L = International units per liter; mL/min/1.73 m² = milliliters per minute per 1.73 meters squared; mOsm/kg H₂O = milliosmoles per kilogram of water

Complete Metabolic Profile	Units and Abbreviations	Normal Range	Patient Value
Sodium*	mEq/L	135–145	126
Potassium	mEq/L	3.5–5.5	4.3
Chloride	mEq/L	96–106	97
Bicarbonate	mEq/L	22–26	25
Anion Gap	mEq/L	4–12	4
Blood urea nitrogen (BUN)*	mg/dL	7–20	21
Creatinine	mg/dL	0.6–1.1	0.85
BUN/creatinine ratio*	Ratio	10:1–20:1	24.7
Glucose	mg/dL	70–99	99
Estimated glomerular filtration rate (eGFR)*	mL/min/1.73 m²	> 90	66
Osmolality calculation	mOsm/kg H₂O	275–295	265
Calcium	mg/dL	8.5–10.5	8.9
Alanine transaminase (ALT)	IU/L	7–55	19
Aspartate transaminase (AST)	IU/L	8–48	14
Alkaline phosphatase (ALP)	IU/L	40–130	44
Bilirubin total	mg/dL	0.1–1.2	0.5
Lactic dehydrogenase units per liter*	IU/L	140–280	283

**Figure 1:**
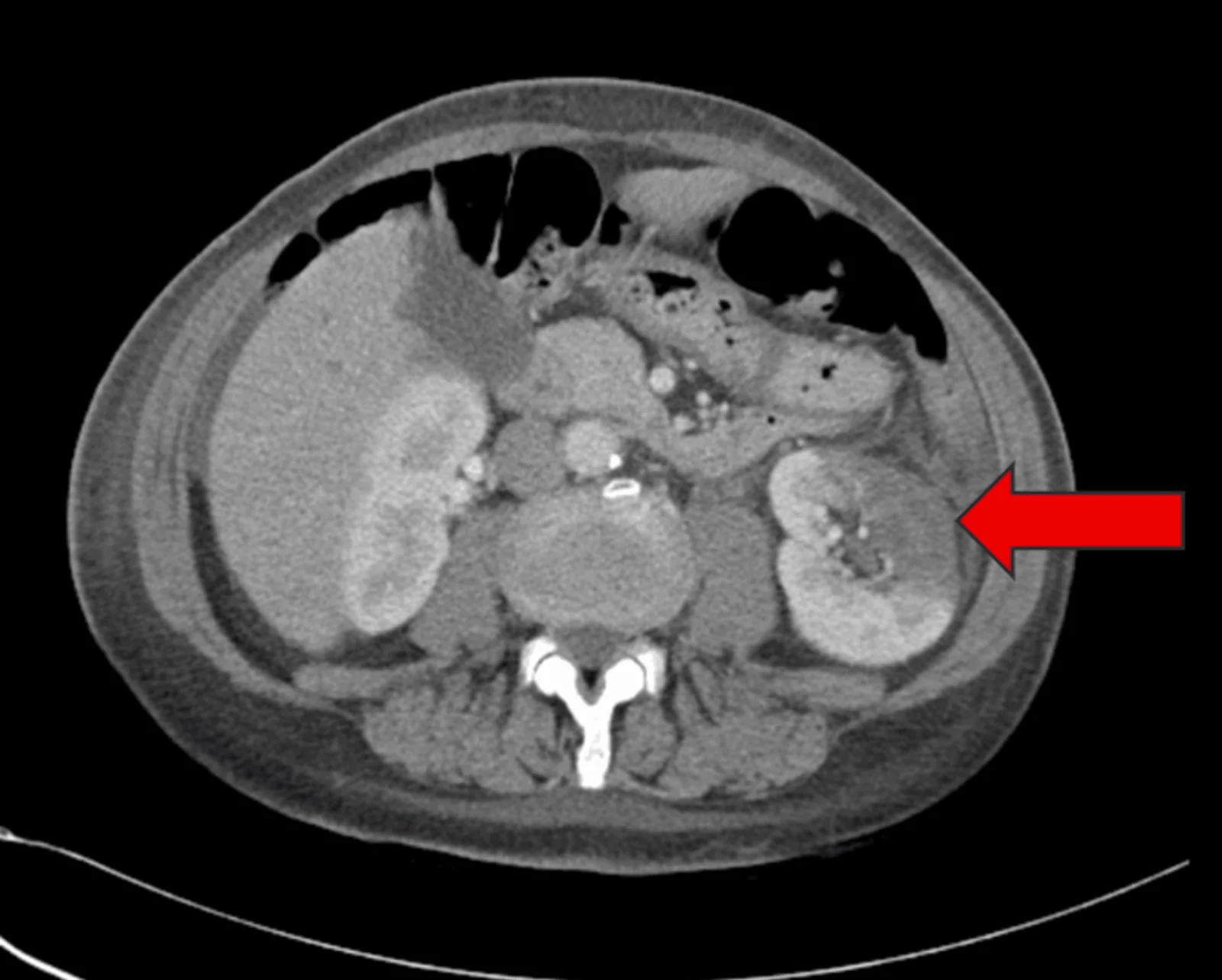
Acute interpolar left renal infarct at the red arrow

Her iron studies were suggestive of inflammation, with ferritin 516.7 ng/mL (nanograms/milliliter) (normal range: 10-150 ng/mL for women) and serum iron 23 µg/dL (micrograms/deciliter) (normal range for women: 60-140 µg/dL). Contrast-enhanced CT demonstrated an acute interpolar left renal infarction with greater than 50% stenosis at the renal artery origin (Figure 1).

The differential diagnosis for this finding included obstructive septic embolus, local mycotic aneurysm formation, or chronic atherosclerotic vascular disease. The initial infectious workup showed negative blood cultures (one set) and negative serologies for HIV, syphilis, hepatitis B, hepatitis C, and hepatitis A (Table 3).

**Table 3:** Infectious workup and serologies

Microbiology and Serology	Reference	Results
Blood cultures	Negative	Negative
*Bartonella henselae* IgG (titer)	<1:64	1:32768
cfDNA *Bartonella* MPM (Molecule per milliliter)	Not detected	29,648
HIV Ag/Ab	Negative	Negative
Treponemal antibody	Negative	Negative
Hepatitis B	Negative	Negative
Hepatitis C	Negative	Negative
Hepatitis A	Negative	Negative
Q fever serology	Negative	Negative
*Brucella* IgG	Negative	Negative
*Brucella* IgM	Negative	Negative
*T. whipplei* PCR	Negative	Negative

**Figure 2:**
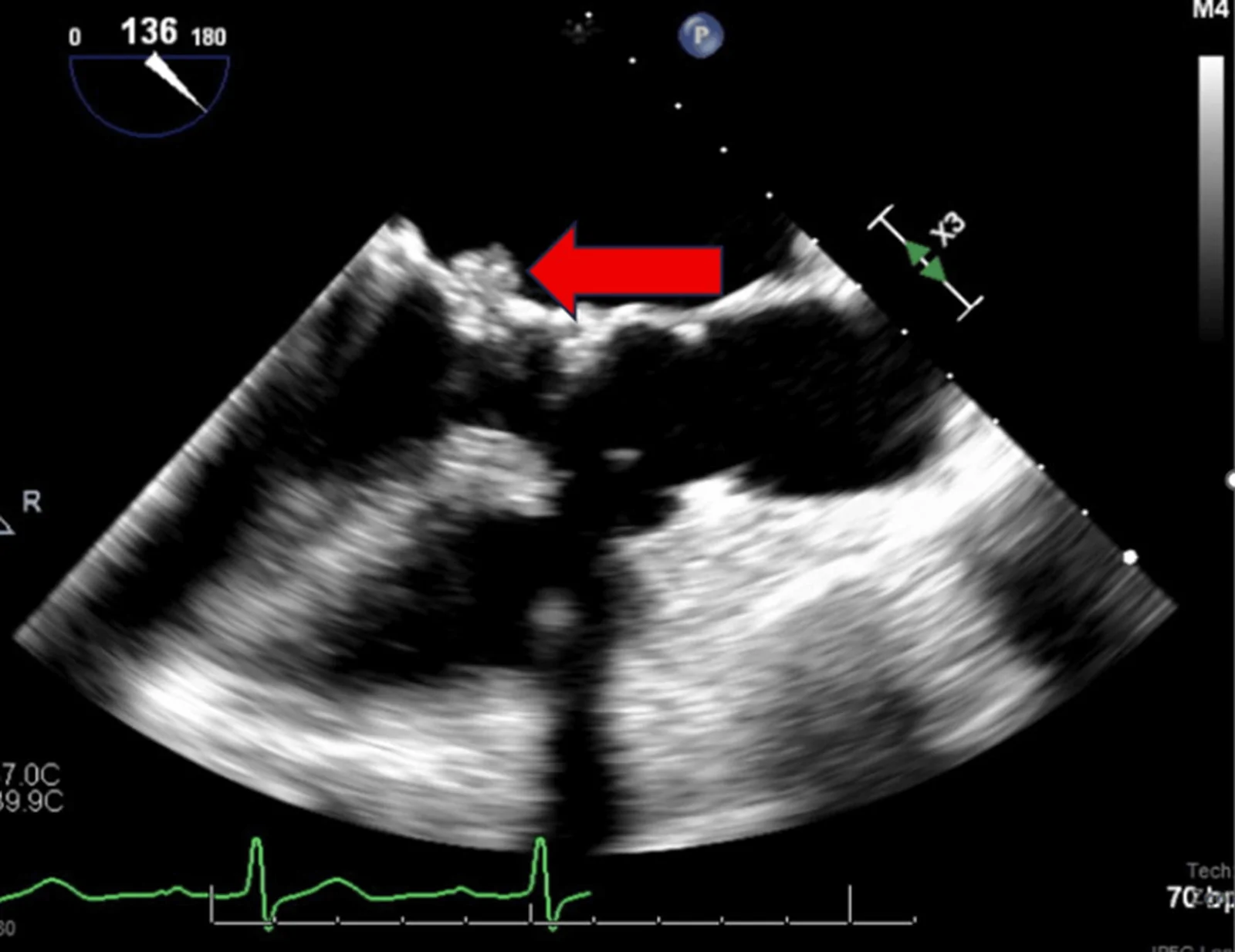
Initial transesophageal echocardiogram (TEE), mid-esophageal four-chamber view, demonstrating irregular thickening of a bioprosthetic mitral valve with a mobile, filamentous density (~10 mm), concerning for vegetation (red arrow)

Transesophageal echocardiography revealed an open left atrial appendage without thrombus and irregular thickening of the bioprosthetic mitral valve with mobile, filamentous densities measuring about 10 mm, concerning for vegetation and prompting evaluation for culture-negative endocarditis (Figure 2).

Given her exposure to wild and feral animals and the chronicity of symptoms, zoonotic infection, including *Bartonella henselae*, *Coxiella burnetii*, and *Brucella* species, was considered, along with culture-negative endocarditis.

Q fever serologies and *Brucella* IgG/IgM were negative. In contrast, *Bartonella henselae* IgG titers were markedly elevated at 1:16384, and microbial cell-free DNA testing detected 29,648 DNA molecules/µL, supporting an active *Bartonella henselae* infection.

**Figure 3:**
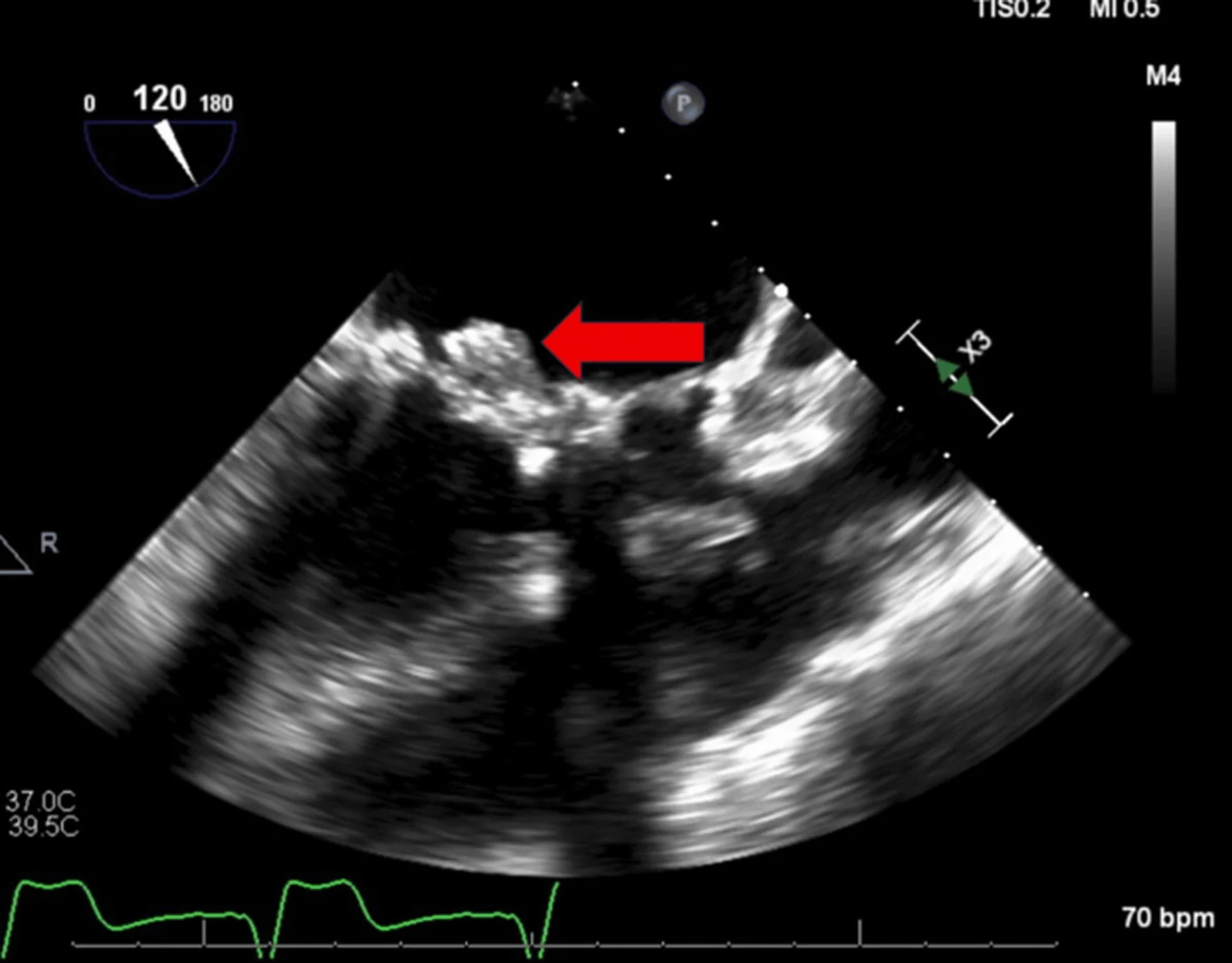
Follow-up transesophageal echocardiogram (TEE), mid-esophageal four-chamber view, performed 14 days after the initial study, demonstrating interval enlargement of a multilobulated, mobile density on a bioprosthetic mitral valve, increasing from ~10 mm to ~16 mm (red arrow)

She was treated with doxycycline 100 mg twice daily plus intravenous gentamicin 1 mg/kg twice daily for two weeks. Cardiothoracic surgery recommended medical management and reassessing the vegetation with a repeat TEE after two weeks of antibiotics. Repeat TEE after two weeks showed enlargement of the mitral vegetation to 16 mm and new mobile densities on a permanent pacemaker lead in the right atrium, which was consistent with a specific, fastidious, facultative intracellular pathogen (Figure 3).

Cardiothoracic surgery subsequently performed a redo sternotomy with mitral valve replacement (29 mm Mosaic), left atrial appendage ligation, aortic valve replacement (21 mm Edwards Inspiris Resilia), and pacemaker replacement. Operative findings showed a friable mitral vegetation near the aortic annulus; severe aortic insufficiency identified intraoperatively prompted aortic valve replacement. Valve pathology showed acute and chronic inflammation without identifiable organisms. Specialized histochemical stains, including Warthin-Starry silver staining, or tissue-based broad-range 16S ribosomal RNA polymerase chain reaction were performed and were negative.

Due to severe acute-on-chronic normocytic anemia, she underwent two bone marrow biopsies that showed hypercellular marrow with myeloid hyperplasia, reactive plasmacytoid lymphocytes, and preserved trilineage hematopoiesis without dysplasia. Cytogenetic and myelodysplastic syndrome FISH studies were negative (Table 4).

**Table 4:** FISH and myelodysplastic panel studies revealed negative cytogenetic and myelodysplastic syndromes FISH = Fluorescence In Situ Hybridization

Test	Result
Monosomy 5	Not Detected
Monosomy 7	Not Detected
Trisomy 8	Not Detected
Trisomy 19	Not Detected
Del(5q), Del(7q), Del(17p)	Not Detected
ETV6 rearrangement	Not Detected
KMT2A rearrangement	Not Detected
FLT3 mutation	Not Detected
FLT3-ITD	Not Detected
FLT3-TKD	Not Detected

These findings raised concern for pure red cell aplasia secondary to infection-related marrow suppression. She was treated with red blood cell transfusions, epoetin alfa, and cyclosporine. The clinical and laboratory findings were also consistent with severe anemia of chronic inflammation due to prolonged systemic infection. The cyclosporine dose was later reduced from 200 mg to 100 mg twice daily because of myalgias.

After two weeks of doxycycline and gentamicin, she was transitioned to doxycycline monotherapy, which was continued for one year, coordinated to end with cyclosporine therapy, with the intent to reduce the recurrence of infection while on an immunosuppressive medication.

*Bartonella henselae* titers were monitored during treatment and declined over time, with IgG titers decreasing from 1:32k to 1:8k about four months after surgery. Although valve pathology did not identify organisms, the marked serologic elevation, microbial cell-free DNA results, and chronic valvular inflammation supported the diagnosis of *Bartonella*-associated infective endocarditis.

After discharge, she was closely followed by hematology/oncology, with temporary twice-weekly CBC monitoring. Her hemoglobin gradually improved on cyclosporine, and a repeat bone marrow biopsy about one year later showed resolution of hypercellularity, with only mild macrocytic normochromic anemia.

## Discussion

This case highlights two key points: *Bartonella* can cause multi-organ disease through dissemination and septic embolization, and its indolent course often delays diagnosis and treatment.

Although bartonellosis can affect nearly any organ system [5], this patient had involvement of the heart, kidneys, brain, and bone marrow.

Bartonellosis is often indolent and difficult to detect, partly because *Bartonella* persists within endothelial cells and erythrocytes, leading to prolonged bacteremia and delayed diagnosis [5-7].

Recognized risk factors for *Bartonella* blood culture-negative endocarditis include cat exposure, structural valvular disease, and homelessness [8]. This patient had cat exposure and preexisting valvular disease.

Our patient presented with fever of unknown origin, blood culture-negative endocarditis, and renal, cerebral, and bone marrow involvement. Bone marrow disease is uncommon and is more often reported in children [9]. In disseminated bartonellosis, however, marrow involvement with reactive hypercellularity may occur, as in this case [10].

Her severe normocytic anemia and marrow findings suggested infection-related pure red cell aplasia, which improved with transfusions, epoetin alfa, and cyclosporine. A repeat biopsy after one year showed resolution.

*Bartonella* is a well-known cause of blood culture-negative endocarditis [11]. Compared with previously reported cases, this patient had more extensive systemic involvement and required replacement of both the mitral and aortic valves. Renal involvement in *Bartonella* infection typically presents as glomerulonephritis and can mimic autoimmune disease. ANCA positivity and hypocomplementemia are frequently reported in *Bartonella* endocarditis-associated glomerulonephritis [12,13], consistent with this patient’s low complement levels and positive c-ANCA. Her renal injury also included an embolic infarction, a rare but reported complication [14]. Neurologic manifestations of *Bartonella* infection more commonly include encephalitis, neuroretinitis, meningitis, and cognitive changes; stroke is uncommon [15]. Her prior left frontal infarction may have been an early embolic complication of evolving endocarditis, although atrial fibrillation remained an alternative explanation.

Cats are the natural reservoir for *B. henselae*, and transmission usually occurs through scratches or bites [16]. Older adults may present atypically and experience longer diagnostic delays, which likely contributed to this patient’s prolonged course [17]. Diagnosis remains challenging because culture, serology, and PCR have limited sensitivity, cross-reactivity, and variable standardization. Newer antigen-based and molecular assays may improve diagnostic accuracy [18].

Treatment of prosthetic valve infection generally requires prolonged combination antimicrobial therapy and adequate source control. In this case, therapy was extended because of dual prosthetic valves, cyclosporine-induced immunosuppression, and the high risk of recurrence after multiple sternotomies [19].

It is important to differentiate proven *Bartonella* tissue involvement from secondary clinical manifestations. In particular, the relationship between the bone marrow abnormalities and *Bartonella* infection remains inferential rather than directly demonstrated.

Another important element regarding the past history is that, given the patient's well-documented history of paroxysmal atrial fibrillation, sick sinus syndrome, and a permanent pacemaker, definitively assigning the stroke to septic embolization without neurovascular histopathology or a clear temporal correlation is speculative. Cardiogenic thromboembolism should be included in the differential diagnosis.

Overall, this case highlights how *Bartonella henselae* can persist, evade early detection, and imitate noninfectious diseases while leading to progressive multisystem complications. It should be considered in the differential diagnosis of culture-negative endocarditis, particularly in patients with cat exposure and valvular risk factors.

## Conclusions

Culture-negative endocarditis caused by *Bartonella henselae* presents a diagnostic challenge due to the lack of standardized criteria. Almost every organ system can be affected, often leading to delayed diagnosis and treatment. This patient's case illustrates the diverse and variable manifestations of bartonellosis and highlights the importance for healthcare providers to maintain a high level of suspicion.
